# The origin of long-chain fatty acids required for de novo ether lipid/plasmalogen synthesis

**DOI:** 10.1016/j.jlr.2023.100364

**Published:** 2023-03-28

**Authors:** Serhii Chornyi, Rob Ofman, Janet Koster, Hans R. Waterham

**Affiliations:** 1 Amsterdam UMC - University of Amsterdam, Department of Clinical Chemistry, Laboratory Genetic Metabolic Diseases, AZ Amsterdam, the Netherlands; 2 Amsterdam Gastroenterology Endocrinology Metabolism, Amsterdam, the Netherlands; 3 Amsterdam Reproduction & Development, Amsterdam, the Netherlands

**Keywords:** Phospholipids, Biosynthesis, Metabolism, Zellweger syndrome, Fatty acid, Transport

## Abstract

Peroxisomes are single-membrane bounded organelles that in humans play a dual role in lipid metabolism, including the degradation of very long-chain fatty acids and the synthesis of ether lipids/plasmalogens. The first step in de novo ether lipid synthesis is mediated by the peroxisomal enzyme glyceronephosphate O-acyltransferase, which has a strict substrate specificity reacting only with the long-chain acyl-CoAs. The aim of this study was to determine the origin of these long-chain acyl-CoAs. To this end, we developed a sensitive method for the measurement of de novo ether phospholipid synthesis in cells and, by CRISPR-Cas9 genome editing, generated a series of HeLa cell lines with deficiencies of proteins involved in peroxisomal biogenesis, beta-oxidation, ether lipid synthesis, or metabolite transport. Our results show that the long-chain acyl-CoAs required for the first step of ether lipid synthesis can be imported from the cytosol by the peroxisomal ABCD proteins, in particular ABCD3. Furthermore, we show that these acyl-CoAs can be produced intraperoxisomally by chain shortening of CoA esters of very long-chain fatty acids via beta-oxidation. Our results demonstrate that peroxisomal beta-oxidation and ether lipid synthesis are intimately connected and that the peroxisomal ABC transporters play a crucial role in de novo ether lipid synthesis.

## Graphical abstract

**Figure:**
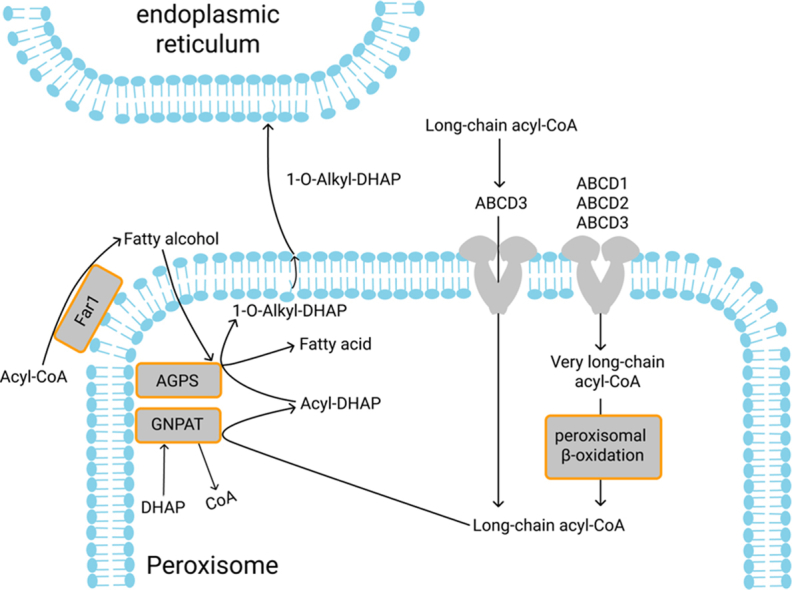

Ether lipids are glycero- and glycerophospholipids in which the hydrocarbon chain at the sn-1 position is attached to the glycerol backbone by an ether bond. Two types of ether lipids are generally discriminated, including plasmanyl, which contain an ether bond, and plasmenyl, which contain a vinyl-ether bond at the sn-1 position. Plasmenyl-phospholipids, also known as plasmalogens, are by far the most abundant ether lipids and represent nearly 18% of all phospholipids in humans (1).

The first steps of de novo synthesis of ether lipids occur in peroxisomes and are mediated by the intraperoxisomal enzymes glyceronephosphate O-acyltransferase (GNPAT, also known as DHAPAT) and alkylglycerone phosphate synthase (AGPS, also known as ADHAPS) and by two enzymes localized at the outer leaflet of the peroxisomal membrane, Fatty acyl-CoA reductase 1 and 2 (FAR1, FAR2). GNPAT catalyzes the conversion of dihydroxyacetone phosphate (DHAP) and acyl-CoA into acyl-DHAP and CoA (2). AGPS subsequently catalyzes the substitution of the acyl chain with a long-chain fatty alcohol resulting in the formation of alkyl-DHAP and the release of the fatty acid (3). The long-chain fatty alcohol required for this reaction is produced from an acyl-CoA on the outer side of the peroxisomal membrane by FAR1 or FAR2 (4). Owing to their hydrophobic properties, the long-chain fatty alcohols are most likely transported across the peroxisomal membrane in a protein-independent manner via a flip-flop mechanism. Alkyl-DHAP is subsequently transported to the endoplasmic reticulum (ER), where the final steps in the formation of mature ether lipids occur (1, 5).

Previously, it was shown that GNPAT has a strong substrate specificity and can only react with hexadecanoyl- (C16:0), tetradecanoyl- (C14:0), cis- or trans-9-hexadecenoyl- (C16:1) CoA esters (2). So far, however, it has remained unknown whether these long-chain acyl-CoAs are produced inside peroxisomes or imported into peroxisomes from the cytosol and which enzymes and transporters are involved. The aim of this study was to determine the origin of the long-chain acyl-CoAs required in the enzyme reaction catalyzed by GNPAT.

In addition to their role in ether lipid synthesis, human peroxisomes play an important role in the degradation of very long-chain, dicarboxylic, and branched-chain fatty acids via beta- or alpha-oxidation (6). Prior to peroxisomal import, these fatty acids are converted to their corresponding CoA esters by extraperoxisomal acyl-CoA synthetases, after which they become a substrate for the peroxisomal transporter proteins that belong to the ATP-binding cassette transporters subfamily D (ABCD). These include three different ABCD transporters, ABCD1 (ALDP, adrenoleukodystrophy protein), ABCD2 (ALDRP, adrenoleukodystrophy-related protein), and ABCD3 (PMP70, 70 kDa peroxisomal membrane protein), which previously were shown to have preferred but overlapping substrate specificities (reviewed in 7). Long-, medium- and short-chain fatty acids are degraded predominantly by mitochondrial beta-oxidation (8). However, when mitochondrial beta-oxidation is defective, long- and medium-chain fatty acids can be degraded in peroxisomes in an ABCD3-dependent manner (9). In the yeast *Saccharomyces cerevisiae,* which does not contain a mitochondrial beta-oxidation system and thus completely relies on peroxisomal beta-oxidation, the long-chain fatty acid tetradecanoic acid is imported into peroxisomes predominantly as free fatty acid (10), and it can also be imported as CoA ester by the yeast ABCD transporters (11, 12). Whether long-chain fatty acids, such as hexadecanoic and tetradecanoic acids, can also pass the human peroxisomal membrane as free fatty acids is not known.

In addition to peroxisomal import, hexadecanoic and tetradecanoic acid are generated inside peroxisomes as products of very long-chain fatty acid beta-oxidation. During peroxisomal beta-oxidation, CoA esters of saturated, mono- or polyunsaturated very long-chain fatty acids are chain-shortened with two carbons per oxidation cycle (6). Results from experiments with purified peroxisomes have shown that the concentration and chain length of the available acyl-CoA esters define after how many cycles peroxisomal beta-oxidation halts (13). The acyl-CoAs produced are then exported out of peroxisomes as carnitine esters or free fatty acids and undergo further oxidation by the mitochondrial beta-oxidation system (6).

To study the contribution of imported and intraperoxisomal-generated hexadecanoic and tetradecanoic acids in the peroxisomal ether lipid synthesis steps and the involvement of different proteins therein, we developed a sensitive LC-MS-based method that allows one to study de novo ether phospholipid synthesis. Using this method, we studied the de novo ether phospholipid synthesis in a series of HeLa cell lines in which we introduced selected single or multiple knockouts of genes encoding proteins with a potential role in peroxisomal ether lipid synthesis using the CRISPR-Cas9 genome editing technology. The selected genes encode 1) the peroxisomal ABC transporters ABCD1, ABCD2, and ABCD3; 2) the beta-oxidation enzymes Acyl-CoA Oxidase 1 and D-bifunctional protein; 3) the ether lipid synthesis enzymes FAR1 and AGPS; and 4) the peroxisomal biogenesis proteins PEX1 and PEX7. Our combined results show that the long-chain acyl-CoA esters required for the first step of the ether lipid synthesis are imported into human peroxisomes from the cytosol by the peroxisomal ABCD transporters, in particular by the ABCD3 protein, and are also produced during the beta-oxidation-mediated chain shortening of very long-chain fatty acids inside peroxisomes.

## MATERIALS AND METHODS

### Cell culture

HeLa cells were routinely cultured at 37°C under an atmosphere of 5% CO_2_ in Dulbecco's modified Eagle's medium (DMEM, high glucose, Gibco) supplemented with 10% fetal bovine serum (Capricorn Scientific, FBS-12A), 25 mM Hepes buffer (VWR), and antibiotics [100 U/ml penicillin (Gibco), 100 μg/ml streptomycin (Gibco), and 250 ng/ml Amphotericin B (Gibco)].

### *De novo* ether phospholipid synthesis

A total of 4∗10^5^ cells were plated at 50%–60% confluence and allowed to adhere overnight at 37°C under an atmosphere of 5% CO_2_. To assess de novo ether phospholipid synthesis, the cell culture medium was supplemented with 40 μM of 1-heptadecanol (98%, Sigma). When indicated, the cell culture medium was supplemented with 20 μM of batyl alcohol (1-O-octadecyl-rac-glycerol, 99%, Sigma) instead of 1-heptadecanol or with 100 μM of hexadecanoic acid (>99%, Sigma) in addition to 1-heptadecanol. All compounds were dissolved in DMSO. The final concentration of DMSO in the medium did not exceed 1%.

After 24 h of incubation, cells were washed twice with phosphate-buffered saline (PBS) and once with 0.9% NaCl solution, scraped from the bottom of the flask and homogenized on ice using a tip sonicator. Protein concentrations were measured using the bicinchoninic acid assay. A mixture of internal standards, including 20 nmol of PC(14:0)_2_ and 5 nmol of PE(14:0)_2_ dissolved in chloroform/methanol (1:1, v/v), and 1.5 ml of chloroform/methanol were added to 150–200 μg of protein of the cell homogenates. The mixture was sonicated in a water bath at room temperature for 10 min, followed by centrifugation at 16,000 *g* for 10 min. The liquid phase was transferred into glass vials and evaporated under a stream of nitrogen at 50°C. The residue was dissolved in 60 μl of chloroform/methanol (1:1, v/v), and 10 μl of the solution was injected into the HPLC-MS system.

The HPLC system consisted of an Ultimate 3000 binary HPLC pump, a vacuum degasser, a column temperature controller, and an autosampler (Thermo Scientific, Waltham, MA). The column temperature was maintained at 35°C. The samples were applied on a Luna Silica 250∗2 mm, 5 μm column (Phenomenex, Torrance, CA). The phospholipids were separated by a linear gradient between solution B (chloroform/methanol, 97:3, v/v) and solution A (methanol/water, 85:15, v/v). Solution A contained 0.25 ml formic acid and 0.5 ml 25% (v/v) aqueous ammonia per liter of eluent, and solution B contained 0.25 ml formic acid per liter of the eluent. The gradient (0.3 ml/min) was as follows: 0–1 min, 10% A; 1–4 min, 10%–20% A; 4–12 min, 20%–85% A; 12–12.1 min, 85%–100% A; 12.1–14.0 min, 100% A; 14–14.1 min, 100%–10% A; and 14.1–15 min, equilibration with 10% A. All gradient steps were linear, and the total analysis time was 15 min. A Q Exactive Plus (Thermo Scientific) was used in positive electrospray ionization mode. Nitrogen was used as the nebulizing gas. The spray voltage used was 3,500 V and the capillary temperature was 256°C (S-lens RF level, 50; auxiliary gas, 10; auxiliary gas temperature, 300°C; sheath gas, 50; sweep cone gas, 2). Mass spectra of phospholipid molecular species were obtained by continuous scanning from *m/z* 150 to *m/z* 2,000 with a resolution of 280,000. Lipidomic analysis of samples was performed at the Core Facility Metabolomics of the Amsterdam UMC, location AMC, Amsterdam, the Netherlands.

Initial annotation of phospholipids in the first lipidomic dataset was done using an in-house developed pipeline (14). In the following experiments, peak identification was performed using Thermo Scientific Xcalibur version 4.3 software based on retention time and *m/z*. The relative abundance of specific lipids (arbitrary unit) is defined as relative to the corresponding internal standard of the same class (PC(14:0)_2_ used for phosphatidylcholine and PE(14:0)_2_ used for phosphatidylethanolamine lipids) assuming similar responses with respect to the standard and corrected for milligram of protein content of the cell lysates used for the analysis. Although after normalization to the internal standards the abundance could be presented as a concentration, we prefer relative abundance because the response of lipid species in the mass spectrometer may vary considerably depending on their fatty acid composition, and hence their concentration can only be estimated by using a single internal standard per class (semiquantitative). The de novo ether phospholipid synthesis was calculated as an increase in the relative abundance of PE(O-39:7) or PC(O-37:4) in 1-heptadecanol-treated cells in comparison with DMSO-treated cells.

### Generation of gene knockout cells by CRISPR-Cas9 genome editing

To disrupt genes in HeLa cells, we used the CRISPR-Cas9 genome editing technology as previously described (15). We designed and used the following gRNAs for targeting the *PEX1* (AGTATATATAACGCGTCAGC), *PEX7* (TATTGGATCCAGATGAAGCT, TACTAATATTGGATCCAGAT), *ABCD1* (CAGACGGCTGCGGAACGACA, GTCGTTCCGCAGCCGTCTGG), *ABCD3* (GACGGCGCGAAACTCCTCGC), *FAR1* (ATTCAGTATATGTTTTGGTG, TGCTTCTGGAAAAGTTGCTG), *AGPS* (TTGGGCGCGGGCGCGAGCTA, CAAAGCGCGGAGAGCCGCGT), *ACOX1* (TGGGTCCCGATTTCACGAAT, TTCACGAATAGGTACGATAA), or *HSD17B4* (CACCGCTGAGGTTCGACGGG, GTTCGACGGGCGGGTGGTAC) genes. The gRNAs were cloned into the pSpCas9(BB)-2A-GFP (15) (Addgene plasmid ID: 48138) plasmid, and cells were transfected using jetPRIME (Polyplus) according to the manufacturer's instructions. On the next day, GFP-positive cells were selected by FACS sorting (SH800 Sony sorter with a 488 nm laser for excitation and a 525/50 nm channel for emission) and seeded one cell per well into 96-well plates. The cells were expanded into clonal cell lines. To verify the gene disruptions, genomic DNA was isolated from the expanded cells, the exons targeted by the gRNAs were PCR-amplified using the Phire Animal Tissue Direct PCR Kit (Thermo Scientific), and the amplicons were Sanger sequenced. Clonal cells with homozygous or compound heterozygous out-of-frame variants were selected, and loss of the protein was confirmed with specific antibodies (see Western blotting). Loss of PEX1 or PEX7 was confirmed based on Sanger sequencing and the mislocalization of PTS1- and/or PTS2-targeted peroxisomal matrix proteins into the cytosol as determined with immunofluorescence microscopy and the lack of processing of the peroxisomal matrix proteins Acyl-CoA oxidase 1 (ACOX1) and/or 3-Ketoacyl-CoA thiolase (ACAA1).

To generate Δ*ABCD1*Δ*ABCD3*, and Δ*ABCD3*Δ*ACOX1* double knock-out cell lines, we disrupted the *ABCD3* or *ACOX1* gene in two independent clonal cell lines with an *ABCD1* or *ABCD3* gene disruption, respectively.

The parental cell line used for the genome editing has been named “wild type” throughout the text.

### Lentiviral plasmids and transduction

The open reading frames of the *ABCD1*, *ABCD2*, or *ABCD3* genes were cloned into the lentiviral vector pLENTI 6.3/TO/V5-DEST (Invitrogen). Hek-293T cells were cotransfected with one of the generated plasmids and the lentiviral packaging plasmids pMD2G, pMDL/RRE, and pRSV/REV using jetPRIME (Polyplus) to produce lentiviruses. Virus-containing media were collected 48 and 72 h after transfection, filtered through 0.45 μm filters, and used to transduce *ΔABCD1ΔABCD3* clone A, *ΔABCD1ΔABCD3* clone B, or *ΔABCD3ΔACOX1* cells. Blasticidin-resistant cells were selected with 3 μg/ml Blasticidin (InvivoGen) for 7–10 days. After selection, cells were allowed to recover for at least 1 week prior to experiments.

### Peroxisomal beta-oxidation

Peroxisomal beta-oxidation of HeLa cells was measured using [1–^14^C]-labeled hexacosanoic acid (C26:0) as substrate (American Radiolabeled Chemical, ARC1253), as described previously (16). Cells (equivalent to 100 μg of protein) were seeded in vials and incubations were started by the addition of a reaction mix containing cell culture medium supplemented with 10 mg/ml α-cyclodextrin, 2 mM carnitine, and 5 μM [1–^14^C] hexacosanoic acid. After 2 h at 37°C, the incubations were terminated by adding perchloric acid; [^14^C]-labeled CO_2_ was collected as described (16). The assay medium was hydrolyzed with NaOH at 50°C for 30 min, and [^14^C]-labeled acid-soluble products were separated from hexacosanoic acid by methanol/chloroform/heptane (1.41/1.25/1.00, v/v/v) extraction and analyzed using a liquid scintillation counter. The sum of end products of beta-oxidation, which includes [^14^C]-labeled CO_2_ and acid-soluble products, was taken as a measure of beta-oxidation activity.

Beta-oxidation-mediated production of long-chain fatty acids was measured after loading cells with deuterium (D)-labeled D_3_-C22:0 (Docosanoic-22,22,22-d3 acid, CDN isotopes, D-5708). Cells were incubated with 10 μM or 30 μM D_3_-C22:0 for 0, 2, 4, 8, 16, 24, or 48 h (17), followed by fatty acid analysis with electrospray ionization tandem mass spectrometry, as previously described (18). Peroxisomal beta-oxidation capacity of cells with lentiviral overexpression of ABCD proteins was measured using D_3_-C22:0 as described above. To this end, cells were incubated for 24 h with 30 μM of D_3_-C22:0, and production of the D_3_-labeled long-chain fatty acids was used to determine the peroxisomal beta-oxidation capacity (17).

### Western blotting

Cell homogenates were prepared by sonication in lysis buffer (50 mM Tris-HCl, 150 mM NaCl, 0.5% sodium deoxycholate, 0.1% SDS, 1% Triton-X100) supplemented with proteases inhibitors (Roche cOmplete). After sonication, LDS Sample Buffer (NuPAGE) and Reducing Agent (NuPAGE) were added, and the samples were heated for 10 min at 70°C. Proteins (15 or 20 μg) were separated on 10% or 4%–12% Bis-Tris precast gels (NuPAGE) and transferred onto nitrocellulose membranes using dry blotting (iBlot 2 system). The PageRuler Unstained Protein Ladder was used as size standards. After blotting, the membranes were blocked with 1% BSA in PBS for 1 h.

Primary antibodies were diluted in Odyssey blocking buffer and used for overnight incubation of the blots. We used anti-ABCD1 (Abcam, ab197013), anti-ABCD2 (GeneTex, [N3C2] GTX112623), anti-ABCD3 (Sigma, SAB4200181), anti-ACOX1 (Abcam, ab184032), anti-DBP/MFP2 (gift from Prof. Takashi Hashimoto (19)), anti-ACAA1 (Sigma, HPA007244), anti-FAR1 (Sigma, HPA017322), anti-AGPS (gift from Dr. Edwin de Vet (20)) antibodies at a 1:2 000 dilution and anti-α-tubulin (Sigma, T6199) as a loading control at a 1:10,000 dilution. For visualization, we used the secondary antibodies IRDye 800 CW goat anti-rabbit or anti-mouse and IRDye 680 RD donkey anti-mouse at a 1:10,000 dilution in PBS, 0.1% Tween-20, 1% goat serum, and the Odyssey Infrared Imaging System (LI-COR Biosciences).

### RNA isolation and sequencing

Total RNA was extracted from cells using Tri Reagent (Sigma) according to the manufacturer's protocol. Genomic DNA contamination was removed with RNase-Free DNase Set (Qiagen) and RNA was further purified with RNeasy MinElute Cleanup Kit (Qiagen). RNA samples were converted to complementary cDNA, and sequencing adapters were added to generate sequencing libraries. Libraries were sequenced at a depth of 40 million reads using the Illumina HiSeq platform and the Kapa mRNA Hyperprep library kit by the Laboratory Genome Diagnostics of the Department of Human Genetics, Amsterdam UMC.

### Statistics and visualization

A one-way ANOVA with Dunnett’s or Tukey’s multiple comparison test was performed to determine significant differences (ns, not significant; ∗*P*-value < 0.05; ∗∗*P*-value < 0.01; ∗∗∗*P*-value < 0.001; ∗∗∗∗*P*-value < 0.0001). We used GraphPad Prism software for statistical analysis and to create the figures. The RNA sequencing data were normalized with DESeq2 and analyzed using R2: Genomics Analysis and Visualization Platform (http://r2.amc.nl). Figma software was used to create the graphical abstract.

## RESULTS

### Development of an assay to measure de novo ether phospholipid synthesis in cells

**Fig. 1:**
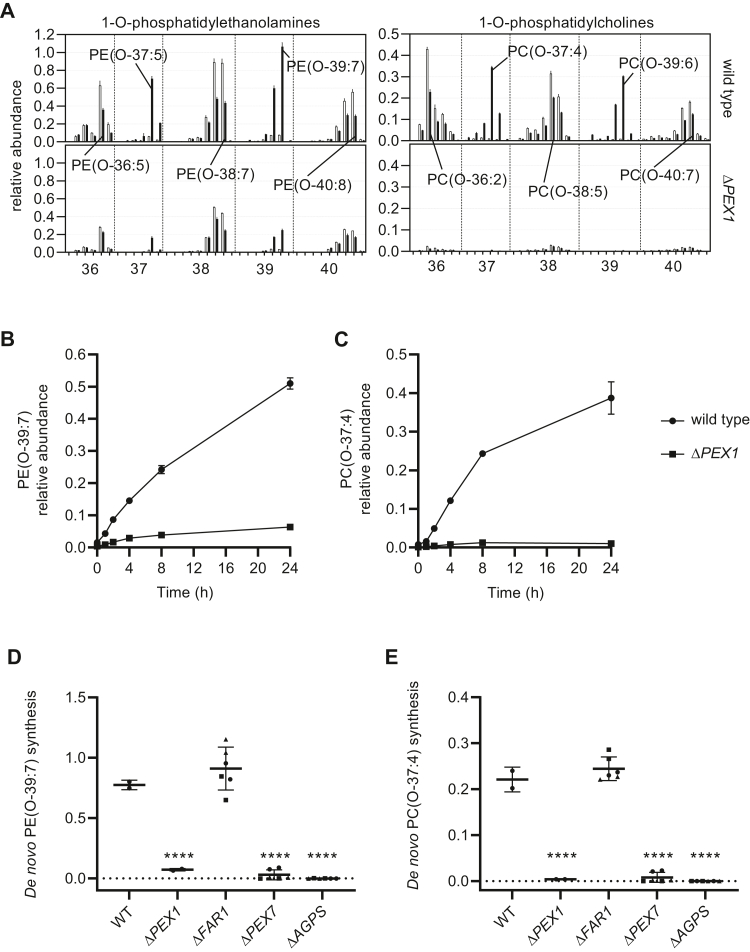
The relative abundance of ether phospholipids with an odd number of side-chain carbons after the incubation of cells with 1-heptadecanol. A: Profiles of phosphatidylethanolamine (PE) and phosphatidylcholine (PC) ether lipids in wild-type and Δ*PEX1* HeLa cells. Cells were cultured with (black bars) or without (white bars) 1-heptadecanol. Ether phospholipids with side chains comprising a total of 36–40 carbon atoms and a different number of double bonds (increases from left to right) are presented. The results are shown as mean ± standard deviation (n = 2). B, C, Time-dependent formation of PE(O-39:7) and PC(O-37:4) in wild-type or Δ*PEX1* HeLa cells after incubation with 1-heptadecanol. The results are shown as mean ± standard deviation (n = 3). D, E, Ether phospholipid synthesis from 1-heptadecanol in wild-type, *ΔPEX1*, *ΔFAR1*, *ΔPEX7*, and *ΔAGPS* HeLa cells. The results are shown as individual values and mean ± standard deviation (n = 2, three different clones are plotted with different symbols). One-way ANOVA with Dunnett’s multiple comparisons test was performed to determine if differences are significant when compared with the wild-type cells. Relative abundance of lipids (arbitrary units) is defined as relative to the corresponding internal standards added prior to lipid extraction (see Materials and Methods section). The de novo ether lipid synthesis of PE(O-39:7) or PC(O-37:4) was calculated as an increase in the relative abundance after 1-heptadecanol treatment (arbitrary units).

Ether lipids most commonly contain a long-chain fatty alcohol with a C16:0, C18:0, or C18:1 hydrocarbon chain at the sn-1 position (5). These long-chain fatty alcohols can be produced from long-chain fatty acids by the peroxisomal enzymes FAR1 or FAR2 or taken up by cells (4, 21, 22). To allow easy identification of *de novo*-produced ether lipids without the use of radio- or isotope-labeled substrates (22), we cultured cells in the presence of the odd-chain long-chain fatty alcohol 1-heptadecanol (C17:0 alcohol) for 24 h and measured the relative abundance of ether phospholipids that incorporated the 1-heptadecanol. Although odd-chain fatty acids and alcohols occur naturally, their abundance is very low in comparison with even-chain fatty acids and alcohols. Because the majority of ether phospholipids have ethanolamine (PE) or choline (PC) as a head group (1), we focused on the de novo synthesis of these ether lipid species. The supplementation of 1-heptadecanol led to the synthesis of multiple ether phospholipid species with an odd number of side-chain carbon atoms and decreased the abundance of virtually all species with an even number of side-chain carbons. When we incubated peroxisome-deficient Δ*PEX1* cells with 1-heptadecanol, we found a marked decrease in the abundance of all ether phospholipids, including the ones with 1-heptadecanol incorporated (Fig. 1A).

In accordance with the fact that most ether phospholipids contain polyunsaturated fatty acids, like C22:6 and C20:4, at the sn-2 position (1), the most abundant ether phospholipids produced de novo from 1-heptadecanol are the plasmanyl-phospholipids O-C37:4 (O-C17:0 at sn-1 and C20:4 at sn-2) and O-C39:6 (O-C17:0 at sn-1 and C22:6 at sn-2), and the plasmenyl-phospholipids O-C37:5 (O-C17:1 at sn-1 and C20:4 at sn-2) and O-C39:7 (O-C17:1 at sn-1 and C22:6 at sn-2) (Fig. 1 A). Ether phospholipids that contain saturated or monounsaturated fatty acids at the sn-2 position (O-C31:0, O-C31:1, O-C33:0, O-C33:1, O-C35:0, O-C35:1) are less abundant (supplemental Fig. S1).

Because the majority of plasmenyl-phospholipids contain an ethanolamine (PE), and plasmanyl-phospholipids an ethanolamine (PE) or choline (PC) as headgroup (1, 23), we selected PE(O-39:7), and PC(O-37:4) as the most representative de novo synthesized ether phospholipids for the two classes. PE(O-39:7) is most likely a plasmenyl-phospholipid, which contains C22:6 at the sn-2 position and C17:1 at the sn-1 position. The double bond at the sn-1 position of PE(O-39:7) is most likely introduced by the plasmanylethanolamine desaturase, which makes it a plasmenyl-phospholipid. PC(O-37:4) is most likely a plasmanyl-phospholipid, which contains C20:4 at the sn-2 position and C17:0 without the additional double bonds at the sn-1 position. Incubation of wild-type HeLa cells with 1-heptadecanol showed a time-dependent increase in both PE(O-39:7) and PC(O-37:4) (Fig. 1B, C), based on which we selected 24 h of incubation for further experiments as it provides higher sensitivity than shorter time points.

**Fig. 2:**
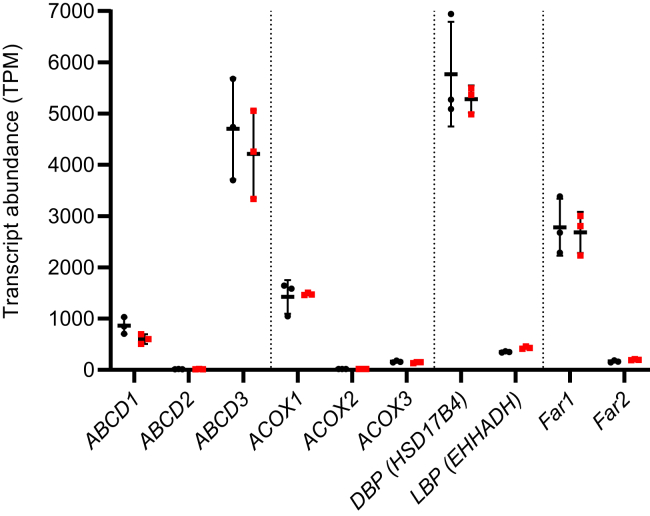
Identification of homologous genes expressed in wild-type (black) or peroxisome-deficient Δ*PEX1* (red) HeLa cells. mRNA abundance (expressed as number of specific transcripts per million transcripts, TPM (24)) of peroxisomal ATP-binding cassette transporters, acyl-CoA oxidases, bifunctional proteins, and fatty acyl-CoA reductases is presented. The results are shown as individual values of three experiments and mean ± standard deviation (n = 3).

We next studied the de novo synthesis in HeLa cells with different gene deletions generated by CRISPR-Cas9 genome editing and resulting in ether lipid deficiencies. As expected, the loss of AGPS or PEX7, encoding the peroxisomal protein receptor for PTS2-targeted proteins, including AGPS, completely blocked the ability of cells to produce ether phospholipids de novo (Fig. 1D, E). Although there is some low expression of *FAR2*, *FAR1* has the highest expression (Fig. 2) and is the main enzyme in HeLa cells that produces long-chain alcohol required for ether lipid synthesis. Accordingly, the loss of FAR1 caused a similar decrease in the endogenous levels of ether phospholipids as the loss of AGPS or PEX7 (supplemental Fig. S2A, B). However, when FAR1-deficient cells are incubated with long-chain alcohol, i.e., the product of FAR1, they were able to produce ether phospholipids (Fig. 1D, E).

As expected, the incubation of cells with 1-heptadecanol also increased the levels of non-ether phospholipids with an odd number of side-chain carbons in a time-dependent manner (supplemental Fig. S3), due to intracellular conversion of 1-heptadecanol to heptadecanoic acid (25), which then is incorporated into such phospholipids. This peroxisome-independent increase in non-ether phospholipids is similar in wild-type and peroxisome-deficient Δ*PEX1* cells.

Taken together, these results show that the incubation of cells with 1-heptadecanol allows one to study the contribution of imported and intraperoxisomal-generated hexadecanoic and tetradecanoic acids in the peroxisomal ether lipid synthesis steps and the involvement of different proteins therein.

### ABCD transporters are required for the import of CoA esters of long-chain fatty acids in human peroxisomes

**Fig. 3:**
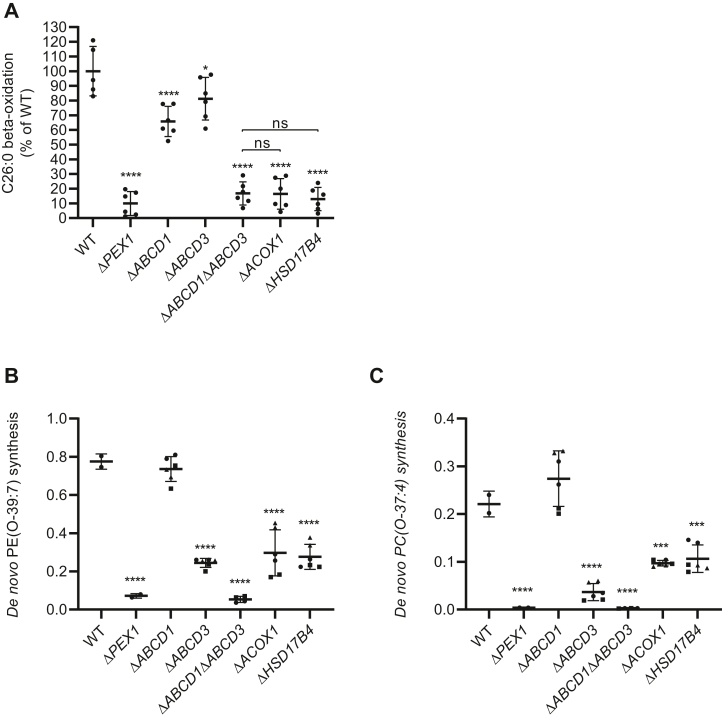
Loss of ABCD transporters or peroxisomal beta-oxidation enzymes causes a decrease in ether phospholipid synthesis in HeLa cells. A: 1-[^14^C] hexacosanoic acid beta-oxidation activity in wild-type, Δ*ABCD1*, Δ*ABCD3*, Δ*ABCD1*Δ*ABCD3*, Δ*PEX1*, Δ*ACOX1*, or Δ*HSD17B4* cells. The results are shown as individual values and mean ± standard deviation. To determine if differences are significant, one-way ANOVA with Tukey’s multiple comparisons test or with Dunnett’s multiple comparisons test when compared with the wild-type cells was performed. Nonsignificant differences are marked as “ns.” B, C, The de novo ether lipid synthesis of PE(O-39:7) or PC(O-37:4) was calculated as an increase in the relative abundance (arbitrary units) (see Materials and Methods section) after 1-heptadecanol treatment. The results are shown as individual values and mean ± standard deviation (n = 2, three different clones are plotted with different symbols). One-way ANOVA with Dunnett’s multiple comparisons test was performed to determine if differences are significant when compared with the wild-type cells.

The peroxisomal ABCD transporters are likely candidates for the import of the long-chain acyl CoA esters required for GNPAT. Of the three known peroxisomal ABCD transporters, ABCD1 and ABCD3, but not ABCD2, are expressed in HeLa cells (Fig. 2). To study the involvement of the ABCD transporters, we disrupted the genes encoding ABCD1 or/and ABCD3. Peroxisomal beta-oxidation activity measurements (Fig. 3A) revealed that hexacosanoic acid beta-oxidation was moderately decreased in the Δ*ABCD1* cells but markedly decreased in the Δ*ABCD1*Δ*ABCD3* cells when compared with the activity in wild-type or Δ*ABCD3* cells, despite ABCD1 being expressed at a much lower level than ABCD3 (Fig. 2). The decrease in the Δ*ABCD1*Δ*ABCD3* cells is similar to the decrease observed in the Δ*PEX1* cells, lacking peroxisomes, and Δ*ACOX1* and Δ*HSD17B4* cells with defective peroxisomal beta-oxidation. These findings are in line with previous studies in yeast that showed that the import of straight-chain acyl-CoA esters into peroxisomes can be mediated by any of the two ABCD transporters, albeit with different efficiency, and that ABCD1 is the predominant transporter for very long-chain acyl-CoA esters (11, 12, 26).

When we next measured the de novo ether phospholipid synthesis in the same cell lines, we found that this was not affected in the Δ*ABCD1*, significantly decreased in the Δ*ABCD3*, and completely deficient in the Δ*ABCD1*Δ*ABCD3* cells (Fig. 3B, C). This indicates that ABCD3 is the predominant transporter of long-chain acyl-CoA esters that are used for ether lipid synthesis. The observation that the ether phospholipid synthesis in the Δ*ABCD1*Δ*ABCD3* cells was decreased to a level similar as in Δ*PEX1* cells (Fig. 3B, C) also indicates that peroxisomal import of long-chain free fatty acids via an ABCD proteins-independent mechanism, as observed in *S. cerevisiae* (10, 11, 12), most probably does not occur in human cells.

Taken together, these results show that, in humans, ABCD transporters are involved and required for the peroxisomal import of the long-chain fatty acids that are used by GNPAT and that ABCD3 is required for normal ether lipid synthesis in HeLa cells.

### Peroxisomal beta-oxidation produces long-chain acyl-CoAs that are required for ether lipid synthesis

The fact that the de novo ether lipid synthesis is not completely deficient in Δ*ABCD3* cells points to the existence of alternative routes for the supply of long-chain acyl-CoAs for the GNPAT reaction. To investigate if the peroxisomal beta-oxidation system could be responsible for providing these long-chain acyl-CoAs, we disrupted the *ACOX1* and *HSD17B4* genes encoding acyl-CoA oxidase (ACOX1) or D-bifunctional protein (DBP/MFP2), respectively, which are the first and second enzyme of the peroxisomal beta-oxidation pathway. As expected, the peroxisomal beta-oxidation in the ACOX1- and DBP/MFP2-deficient cells is as deficient as in the Δ*PEX1* cells (Fig. 3A). When we measured the *de novo* ether phospholipid synthesis in the beta-oxidation-deficient cells, we found it significantly decreased in comparison with the wild-type cells but not as deficient as in the Δ*PEX1* or Δ*ABCD1*Δ*ABCD3* cells (Fig. 3B, C). This strongly suggests that the intraperoxisomal beta-oxidation-mediated shortening of very long-chain acyl-CoAs can supply long-chain acyl-CoAs required for the GNPAT reaction of the de novo ether lipid synthesis pathway.

**Fig. 4:**
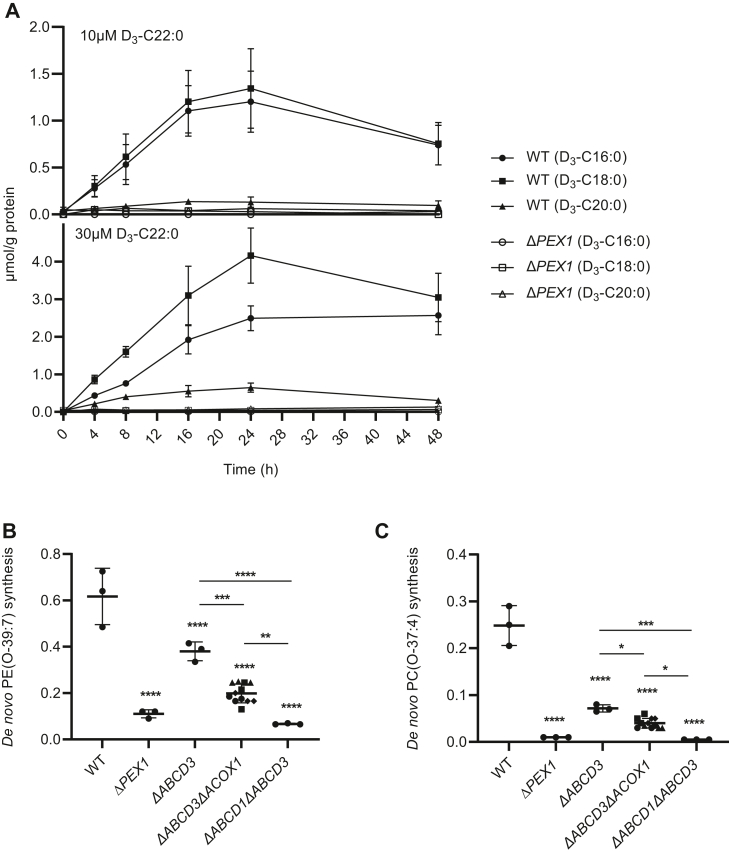
Peroxisomal beta-oxidation supplies long-chain acyl-CoA for GNPAT. (A) Wild-type or Δ*PEX1* cells were incubated with 10 μM or 30 μM of deuterium (D)-labeled D_3_-docosanoic acid. The amount of produced D_3_-labeled hexadecanoic (circle), octadecanoic (square), and eicosanoic (triangle) acids was measured after incubation for the indicated amount of time. The results are shown as mean values ± standard deviation (n = 3). (B–C) Loss of peroxisomal beta-oxidation in Δ*ABCD3* cells leads to a decrease in ether lipid synthesis. The de novo ether lipid synthesis of PE(O-39:7) or PC(O-37:4) was calculated as an increase in the relative abundance (arbitrary units) (see Materials and Methods section) after 1-heptadecanol treatment. The results are shown as individual values and mean ± standard deviation (n = 3, different clones are plotted with different symbols). To determine if differences are significant, one-way ANOVA with Tukey’s multiple comparisons test or with Dunnett’s multiple comparisons test when compared with the wild-type cells was performed.

To confirm that sufficient amounts of long-chain fatty acids are produced by the peroxisomal beta-oxidation of very long-chain fatty acids, we incubated HeLa cells with deuterium-labeled docosanoic acid (D_3_-C22:0) and measured the produced fatty acids with shortened chains. We found that wild-type cells indeed produced deuterium-labeled eicosanoic (D_3_-C20:0), octadecanoic (D_3_-C18:0), and hexadecanoic (D_3_-C16:0) acids, while tetradecanoic acid (D_3_-C14:0) was not detected (Fig. 4A). In D_3_-C22:0-incubated Δ*PEX1* cells very low amounts of these fatty acids were detected, confirming that in wild-type cells they are produced via peroxisomal beta-oxidation (Fig. 4A).

That ABCD3-mediated import of acyl-CoAs and the peroxisomal beta-oxidation-mediated production of long-chain acyl-CoAs form different supply routes for GNPAT also follows from the decreased level of de novo ether phospholipids produced in the Δ*ABCD3*Δ*ACOX1* cells when compared with the Δ*ABCD3* cells (Fig. 4B, C).

### Ether phospholipid synthesis steps mediated by the ER-localized enzymes are not affected in the beta-oxidation-deficient cells

**Fig. 5:**
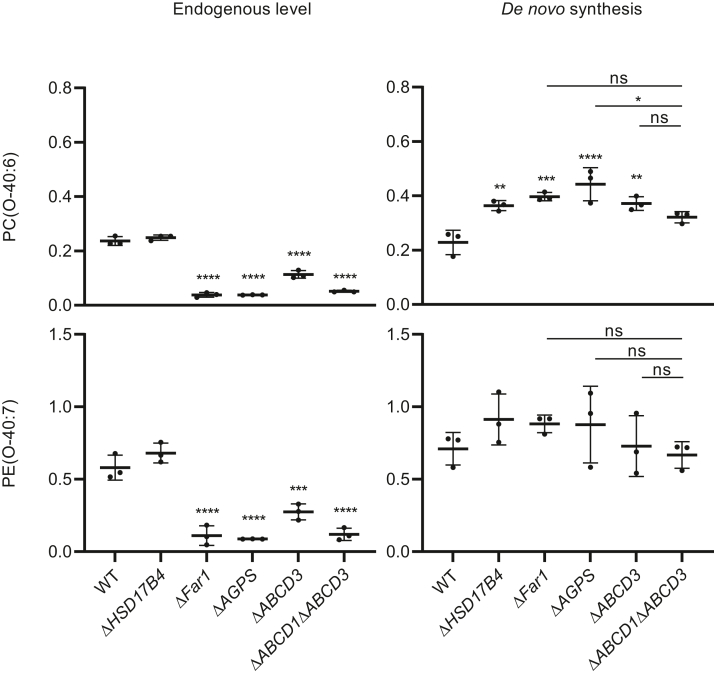
ER-localized ether phospholipid synthesis steps are not affected by peroxisomal beta-oxidation deficiency. Wild-type, Δ*HSD17B4*, Δ*FAR1*, Δ*AGPS*, Δ*ABCD3*, or Δ*ABCD1*Δ*ABCD3* cells were cultured with or without 20 μM of batyl alcohol (1-O-octadecyl-rac-glycerol) for 24 h. The relative abundance of PE(O-40:7) and PC(O-40:6) endogenous (left) and de novo synthesized from batyl alcohol (right) is presented. Relative abundance of lipids is defined as relative to the corresponding internal standards added prior to lipid extraction (arbitrary units) (see Materials and Methods section). The de novo ether lipid synthesis of PE(O-40:7) or PC(O-40:6) was calculated as an increase in the relative abundance after batyl alcohol treatment (arbitrary units). The results are shown as individual values and mean ± standard deviation (n = 3). To determine if differences are significant, one-way ANOVA with Tukey’s multiple comparisons test or with Dunnett’s multiple comparisons test when compared with the wild-type cells was performed.

The final steps of the ether phospholipid synthesis occur in the endoplasmic reticulum. It was previously suggested that the accumulation of very long-chain fatty acids in beta-oxidation-deficient cells can affect ER proteostasis and functions and thus may affect phospholipid synthesis (27, 28). We therefore studied whether the ether phospholipid synthesis steps localized at the ER are affected in the generated beta-oxidation-deficient cells. To this end, we incubated cells defective in beta-oxidation (Δ*HSD17B4*), ether lipid synthesis (Δ*FAR1* and Δ*AGPS*), or both ether lipid synthesis and beta-oxidation (Δ*ABCD3* and Δ*ABCD1*Δ*ABCD3*) with batyl alcohol (1-O-octadecyl-rac-glycerol), which bypasses the peroxisomal steps and can be used directly at the ER for the synthesis of ether lipids (22, 29). After the incubation, the abundance of PC(O-40:6) and PE(O-40:7), products of the de novo synthesis from batyl alcohol (O-C18:0 from batyl alcohol at sn-1 and C22:6 at sn-2), increased more than 2-fold (Fig. 5). The amounts of the ether phospholipids produced de novo were similar between cells with the deficiency of only ether lipid synthesis (Δ*FAR1*, Δ*AGPS*, and Δ*ABCD3*) or both ether lipid synthesis and peroxisomal beta-oxidation (Δ*ABCD1*Δ*ABCD3*), or between the wild-type and beta-oxidation-deficient cells (Δ*HSD17B4*) (Fig. 5). All cells deficient of de novo ether lipid synthesis from 1-heptadecanol produced more de novo PC(O-40:6) from batyl alcohol than the wild-type cells (Fig. 5). Combined, these findings show that the peroxisomal beta-oxidation deficiency does not affect the ether phospholipid synthesis steps localized at the ER.

### Supplementation of hexadecanoic acid rescues the de novo ether lipid synthesis in beta-oxidation-deficient cells

**Fig. 6:**
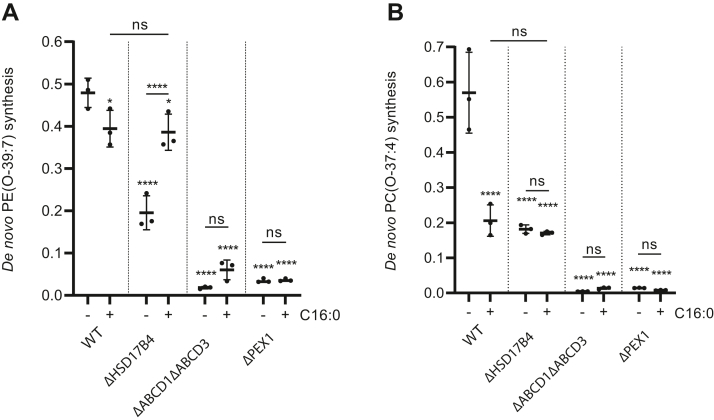
Supplementation of hexadecanoic acid rescues the de novo ether lipid synthesis in beta-oxidation-deficient cells. HeLa cells were cultured with 1-heptadecanol and in addition with or without hexadecanoic acid (100 μM), as indicated. The de novo ether lipid synthesis of PE(O-39:7) (A) or PC(O-37:4) (B) was calculated as an increase in the relative abundance (arbitrary units) (see Materials and Methods section) after 1-heptadecanol treatment. The results are shown as individual values and mean ± standard deviation (n = 3). To determine if differences are significant, one-way ANOVA with Tukey’s multiple comparisons test or with Dunnett’s multiple comparisons test when compared with the wild-type cells was performed.

We showed above that deficiency of both peroxisomal beta-oxidation and ABCD3 results in a peroxisomal de novo ether lipid synthesis defect. We hypothesized that supplementation of cells with hexadecanoic acid may increase the ABCD-mediated import of acyl-CoA into peroxisomes and thus rescue the de novo ether lipid synthesis in beta-oxidation-deficient cells. To test this, cells were supplemented with hexadecanoic acid in addition to 1-heptadecanol. In all cells, the supplementation of hexadecanoic acid had hardly any effect on the abundance of non-ether phosphatidylethanolamine species containing a hexadecanoyl chain but led to an increase in the abundance of non-ether phosphatidylcholine species containing a hexadecanoyl chain (supplemental Fig. S4). Interestingly, while the supplementation caused a small decrease in wild-type cells, it almost fully rescued de novo ether lipid synthesis of ether phosphatidylethanolamine species in the Δ*HSD17B4* cells (Fig. 6A). The rescue is dependent on ABCD transporters, as this increase in ether phosphatidylethanolamine species was not observed in the Δ*ABCD1*Δ*ABCD3* cells (Fig. 6A). For the ether phosphatidylcholine species, including PC(O-37:4), however, the supplementation resulted in a marked decrease in the wild-type cells while no change was observed in the other cells (Fig. 6B).

Combined, these results strongly suggest that the supplementation of the cell culture medium with hexadecanoic acid increases the ABCD-mediated import of long-chain acyl-CoAs into peroxisomes.

### ABCD1 and ABCD2 also import the acyl-CoAs required for de novo ether lipid synthesis

**Fig. 7:**
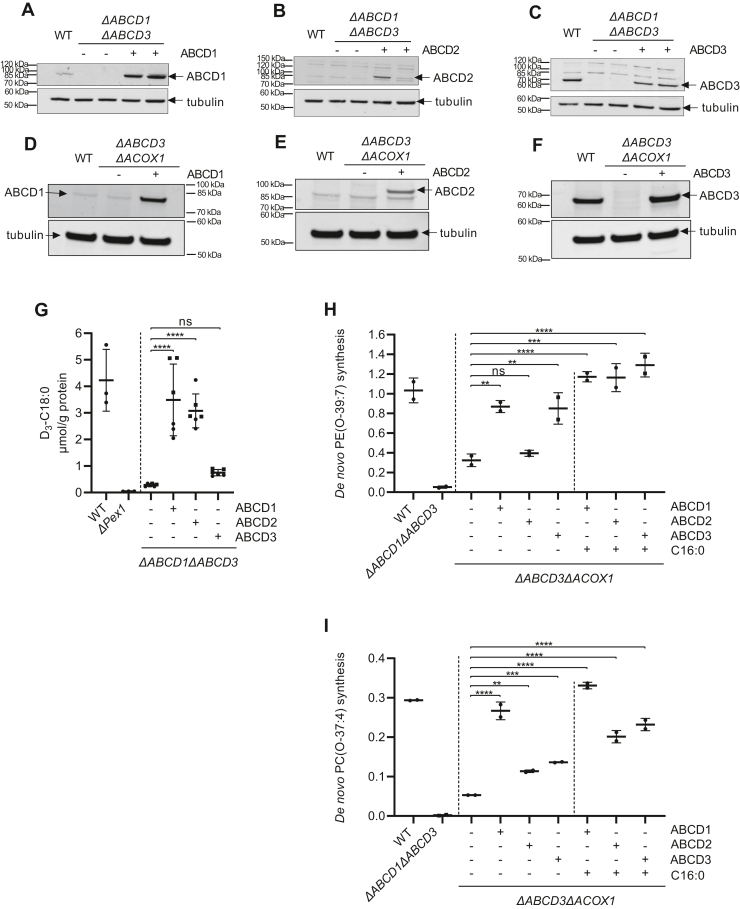
Peroxisomal ABCD transporters can import long-chain fatty acids used in de novo ether lipid synthesis. ABCD1, ABCD2, or ABCD3 was expressed in *ΔABCD1ΔABCD3* cells (A–C) or *ΔABCD3ΔACOX1* cells (D–F). Total cell lysates containing equal amounts of protein were processed by Western blotting with specified antibodies against ABCD1 (A, D), ABCD2 (B, E), or ABCD3 (C, F). The arrows mark specific immune-reactive bands. G: Wild-type, Δ*PEX1*, Δ*ABCD1*Δ*ABCD3*, or Δ*ABCD1*Δ*ABCD3* HeLa cells expressing one of the ABCD transporters were incubated with 30 μM of D_3_-docosanoic acid for 24 h. The amount of produced D_3_-labeled octadecanoic acid was measured. Results are shown as mean values ± standard deviation (n=3, different clones are plotted with different symbols). H–I, Wild-type, Δ*ABCD1*Δ*ABCD3*, Δ*ABCD3*Δ*ACOX1*, or Δ*ABCD3*Δ*ACOX1* cells after expression of one of the ABCD transporters were cultured with 1-heptadecanol and in addition with or without hexadecanoic acid (100 μM), as indicated. The de novo ether lipid synthesis of PE(O-39:7) or PC(O-37:4) was calculated as an increase in the relative abundance (arbitrary units) (see Materials and Methods section) after 1-heptadecanol treatment. The results are shown as individual values and mean ± standard deviation (n = 2). To determine if differences are significant, one-way ANOVA with Tukey’s multiple comparisons test or with Dunnett’s multiple comparisons test when compared with the wild-type cells was performed.

ABCD2 has overlapping substrate specificity with other peroxisomal ABCD transporters but a different tissue and cell expression pattern (11,30) and is not expressed in HeLa cells (Fig. 2). To determine if ABCD2 also can import acyl-CoAs required for GNPAT from the cytosol, and to functionally compare all three ABCD transporters, we stably expressed ABCD1, ABCD2, or ABCD3 protein in the Δ*ABCD3*Δ*ACOX1* cells, which are defective for beta-oxidation and lack the ABCD3 transporter, and in the ΔA*BCD1*Δ*ABCD3* cells, which lack all peroxisomal ABCD transporters. Immunoblot analysis of the different transduced cells revealed high protein levels for all peroxisomal ABCD proteins; the levels of ABCD3 were similar to the endogenous levels in the nontransduced wild-type cells (Fig. 7A–F).

When expressed in the Δ*ABCD1*Δ*ABCD3* cells, both ABCD2 and ABCD1 proteins were able to rescue the beta-oxidation-mediated shortening of D_3_-docosanoic acid to a similar extent, while ABCD3 expression led to only a minor increase in the production of D_3_-long chain fatty acids (Figs. 7G and S5). This confirms that, similar to ABCD1, ABCD2 can efficiently import CoA esters of straight-chain very long-chain fatty acids such as docosanoic acid into peroxisomes (11, 30).

When we then measured the de novo ether phospholipid synthesis in the beta-oxidation-deficient Δ*ABCD3*Δ*ACOX1* cells, however, we found it significantly increased when ABCD1 or ABCD3 is expressed (Fig. 7H, I). This indicates that not only ABCD3 but also ACBD1 imports long-chain fatty acids into peroxisomes. The ability of ABCD1 to import long-chain fatty acids also explains why the Δ*ABCD3*Δ*ACOX1* cells, which still express endogenous ABCD1, produce more ether phospholipids than Δ*ABCD1*Δ*ABCD3* cells (Figs. 4B, C and 7H, I). We also observed that ABCD3 expression rescued the synthesis of PE(O-39:7) to a greater extent than PC(O-37:4).

To stimulate the ABCD proteins-mediated import of long-chain fatty acids, we incubated the different Δ*ABCD3*Δ*ACOX1* cells with hexadecanoic acid, in addition to 1-heptadecanol. This resulted in a further increase in ether phospholipid synthesis in the Δ*ABCD3*Δ*ACOX1* cells expressing not only ABCD1 or ABCD3 but also ABCD2 (Fig. 7H, I). These combined results show that in principle all three peroxisomal ABCD transporters can import long-chain fatty acids into peroxisomes.

## DISCUSSION

We developed a novel assay to study de novo ether phospholipid synthesis in cells. Using the assay we showed that long-chain acyl-CoA molecules required for the first intraperoxisomal step in de novo ether lipid synthesis can be imported into peroxisomes from the cytosol by the peroxisomal ABC transporters, in particular ABCD3, as well as supplied through the beta-oxidation-mediated chain shortening of very long-chain fatty acids inside peroxisomes.

Defects in genes encoding the peroxisomal enzymes involved in ether lipid synthesis or required for their peroxisomal import result in severe autosomal recessive inborn disorders, called Rhizomelic chondrodysplasia punctata type 1 (PEX7 deficiency, OMIM 215100), type 2 (GNPAT deficiency, OMIM 222765), type 3 (AGPS deficiency, OMIM 600121), or disorder reminiscent of Rhizomelic chondrodysplasia punctata (FAR1 deficiency, OMIM 616154). Disease severity of these disorders correlates with the deficiency of ether lipid synthesis and, in most severe cases, the defect results in a short life expectancy (31, 32). Here, we report that, in addition to cells with a primary defect in ether lipid synthesis, cells with a beta-oxidation deficiency, i.e., Δ*ACOX1* or Δ*HSD17B4*, have a decreased de novo ether phospholipid synthesis, albeit less severe. This corroborates previously reported lipidomics results, which revealed a decrease in specific ether lipid species in fibroblasts from patients with single peroxisomal enzyme beta-oxidation deficiencies (28). Moreover, this finding confirms the here reported role of peroxisomal beta-oxidation in providing long-chain acyl-CoAs that can be used for ether lipid synthesis. Of note, the commonly used diagnostic measurement of plasmalogens in erythrocytes or fibroblasts (33) does not reveal a deficiency in patients with single peroxisomal enzyme beta-oxidation deficiency, which may be attributed to the sensitivity of this assay. In addition, this does not exclude that de novo ether lipid synthesis is deficient in certain tissues of these patients.

Peroxisomal beta-oxidation of very long-chain fatty acids is known to be incomplete as it results in only partial shortening of the acyl chain (6, 34, 35). Substrate specificity of peroxisomal carnitine O-octanoyltransferase (36), ACOX1 (37), and in vitro experiments with purified peroxisomes (13) have indicated that the peroxisomal beta-oxidation eventually may produce hexanoyl- (C6:0) or octanoyl- (C8:0) CoA. Here we showed that hexadecanoic and octadecanoic acids are produced during the peroxisomal beta-oxidation. The amount of these fatty acids produced from docosanoic acid increases linearly with time, indicating that in vivo a part of the hexadecanoyl- and octadecanoyl-CoA does not undergo further cycles of peroxisomal beta-oxidation and can be used to synthesize complex lipids.

We showed that supplementation of culture medium with hexadecanoic acid rescued the de novo ether lipid synthesis in beta-oxidation-deficient cells. However, we also observed that the supplementation lowered the level of ether phosphatidylcholine species in wild-type cells and that the effect thereof in beta-oxidation-deficient Δ*HSD17B4* cells was different for phosphatidylcholine and phosphatidylethanolamine ether lipids (Fig. 6). In fact, the incubation with hexadecanoic acid resulted in increased levels of hexadecanoyl chain-containing non-ether phosphatidylcholine species in wild-type, Δ*HSD17B4*, Δ*ABCD1*Δ*ABCD3*, and Δ*PEX1* cells (supplemental Fig. S4), but, in wild-type cells, in a decrease in phosphatidylcholine species that do not contain a hexadecanoyl chain, including PC(O-37:4). For ether phosphatidylethanolamine species this effect is less clear as we only observe a small decrease in the abundance of ether phosphatidylethanolamine species like PE(O-39:7) in wild-type cells after supplementation with hexadecanoic acid. We do not have a clear explanation for the observed decrease in the de novo ether phosphatidylcholine species in the wild-type cells. One possibility is that this is somehow due to the increased synthesis of hexadecanoyl chain-containing non-ether phosphatidylcholine species. Another possibility could be that the supplemented hexadecanoic acid is converted intracellularly into 1-hexadecanol, which can then compete with the added 1-heptadecanol, causing the reduction in de novo synthesized ether phosphatidylcholine species when compared with incubation with 1-heptadecanol alone. Indeed, after its supplementation to cells, hexadecanoic acid is to some extent converted into 1-hexadecanol, most probably by FAR1, as can be deduced from the increase in abundance of 1-hexadecanol-containing ether (lyso)phospholipids (supplemental Fig. S6). However, we think that this competition has a minor effect because the amount of 1-hexadecanol produced by FAR1 is likely much lower than the amount of added 1-heptadecanol, as is also indicated by the larger increase in abundance of 1-heptadecanol-containing ether (lyso)phospholipids than 1-hexadecanol-containing ether (lyso)phospholipids after the incubation (supplemental Fig. S6). Furthermore, we would expect that 1-hexadecanol incorporates into the same ether lipid species as 1-heptadecanol, and therefore the production of 1-hexadecanol from hexadecanoic acid most likely does not explain the difference between de novo synthesis of phosphatidylcholine and phosphatidylethanolamine ether phospholipids.

Our data indicate that the import of long-chain CoA esters from the cytosol into human peroxisomes completely depends on peroxisomal ABCD transporters, as no de novo ether lipid synthesis was observed in Δ*ABCD1*Δ*ABCD3* cells even after incubation with 100 μM of hexadecanoic acid. In principle, the latter could be explained by the absence of an intraperoxisomal acyl-CoA synthetase able to activate long-chain fatty acids like hexadecanoic acid to the corresponding CoA esters. However, we consider this possibility not very likely. First, because in humans there are multiple cytosolic acyl-CoA synthetases that can activate long-chain fatty acids to CoA esters, we expect that the actual pool of free fatty acids in the cytosol will be very small and also that the hexadecanoic acid that we supplemented to the culture medium is likely rapidly converted into CoA ester before it reaches the peroxisomes. Because CoA esters of fatty acids cannot freely pass the membrane, they subsequently require ABCD transporters for their import into peroxisomes, hence the defect in de novo ether lipid synthesis we observe. Second, even if free hexadecanoic acid would reach the peroxisomal membrane, its diffusion across the membrane will probably be very slow. For comparison, in the yeast *S. cerevisiae* short-, medium-, and some long-chain fatty acids can be imported into yeast peroxisomes as free fatty acids (10). It has been suggested that the peroxisomal protein Pex11p might form a channel that can facilitate this free fatty acid import (38, 39). A probable reason for this free fatty acid peroxisomal import in yeast is that the only acyl-CoA synthetase able to activate these fatty acids, Faa2, is localized inside the peroxisomal lumen (10). Thus, because there is no acyl-CoA synthetase in the cytosol capable of activating these fatty acids, they reach peroxisomes in the unesterified form. Based on this, we conclude that, in contrast to the case in yeast *S. cerevisiae* (10, 11, 12), ABCD-independent import of long-chain fatty acids most probably does not occur in human cells.

Of the three ABCD transporters, the expression of ABCD3 is the highest in HeLa cells, as well as in other cell models used for peroxisomal research, like fibroblasts or Hek293 cells, and brain and liver, which are relevant tissues for de novo ether lipid synthesis and peroxisomal beta-oxidation of fatty acids. As we found that ABCD3 is the main transporter for the long-chain acyl-CoAs used for ether lipid synthesis and the loss of ABCD3 in HeLa cells results in decreased ether lipid synthesis, one would expect that a deficiency of ABCD3 results in a disease characterized by severe ether lipid deficiency, similar as observed in the Rhizomelic chondrodysplasia subtypes. So far, however, only one patient with a genetic defect causing a partial ABCD3 deficiency has been reported (40). As far as known, only the levels of C16- and C18-containing plasmalogens have been studied in patient’s cells, which, based on a semiquantitative ratiometric transmethylation-based approach, were slightly reduced in erythrocytes of the patient (9.8 for C18-containing plasmalogens with a reference range of 10.6–24.9; 3.8 for C16-containing plasmalogens with a reference range of 6.8–11.9) but normal in the fibroblasts (40). The absence of a clear ether lipid deficiency may be due to residual activity of the mutated ABCD3 protein, which was truncated with 24 amino acids at the C terminus. Apart from that, in tissues where the other ABCD transporters are highly expressed, they may take over the function of ABCD3.

## Data availability

This study includes no data deposited in external repositories.

## Supplemental data

This article contains supplemental data.

## Conflict of interest

The authors declare that they have no conflicts of interest with the contents of this article.

## Supplementary data

Figure S6

supplemental figure captions
