# Supplementary figures and images for: The origin of long-chain fatty acids required for de novo ether lipid/plasmalogen synthesis
Source: J Lipid Res. 2023 Mar 28;64(5):100364. doi: 10.1016/j.jlr.2023.100364 (PMC10154978; doi:10.1016/j.jlr.2023.100364)

# 1-O-phosphatidylethanolamines

wild type

$\Delta PEX1$

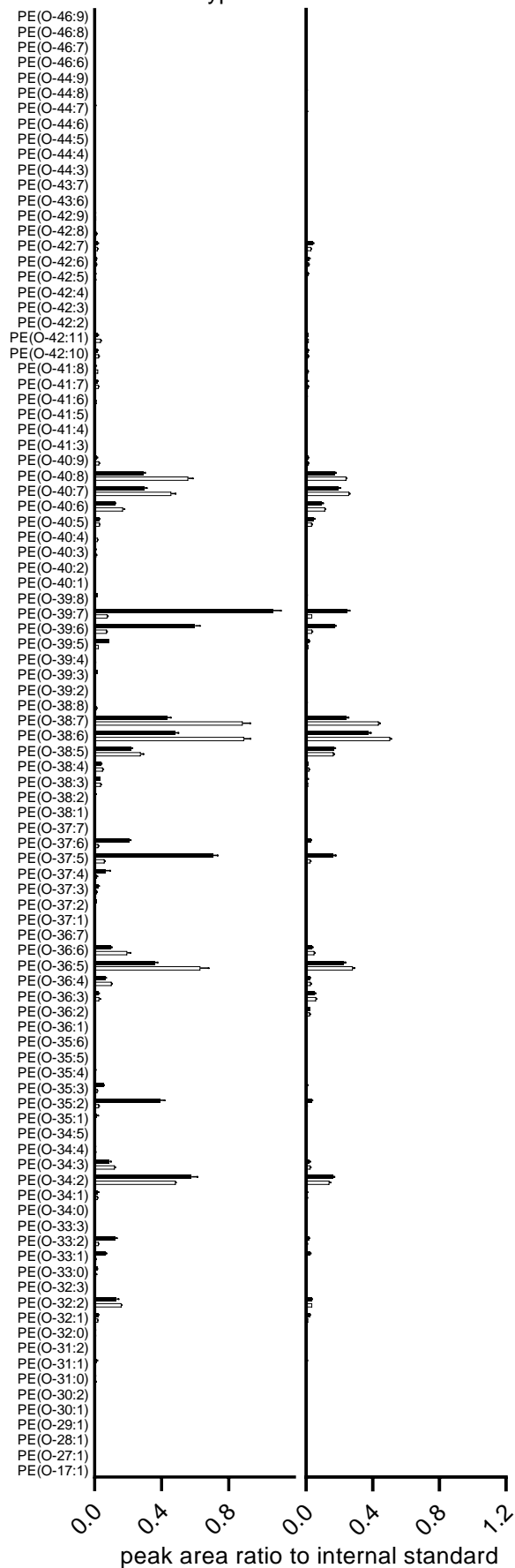

# 1-O-phosphatidylcholines

wild type

$\Delta PEX1$

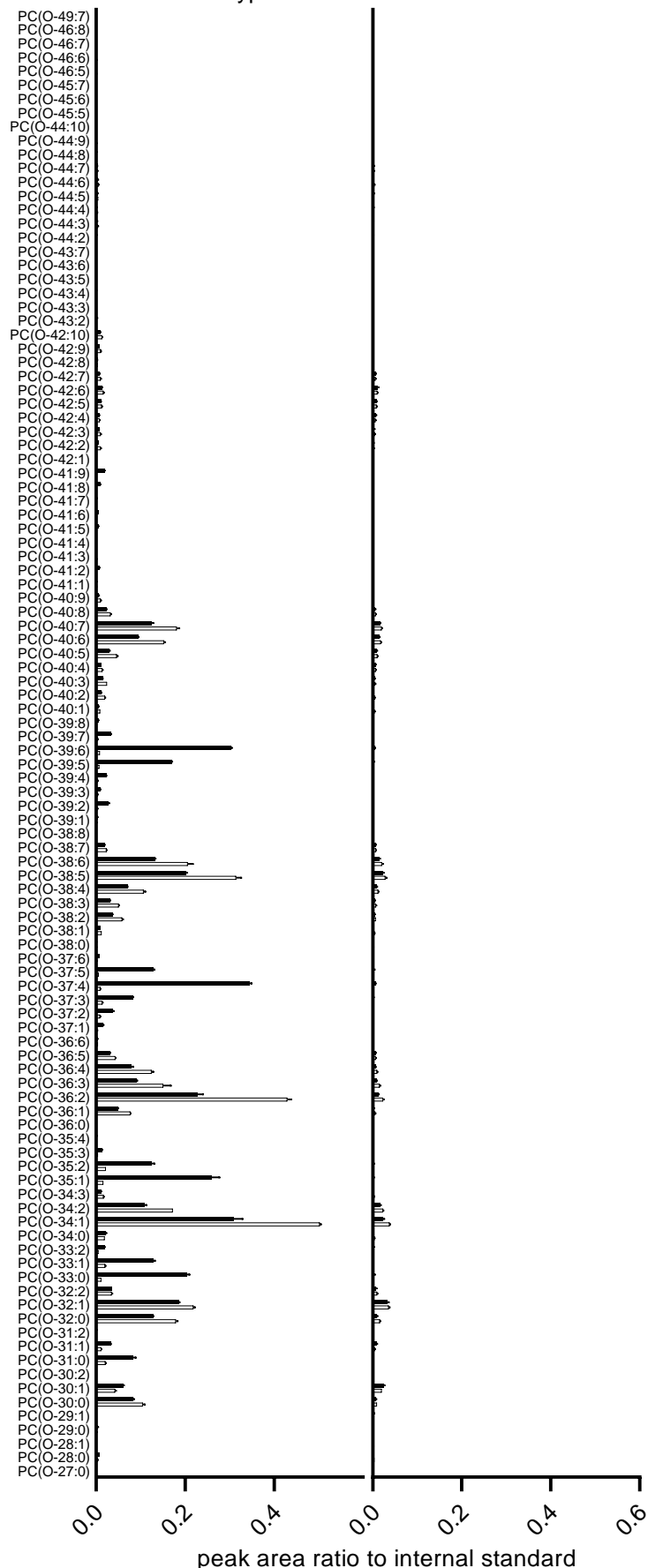

Supplement: Figure S1 [file mmc1.pdf]

A

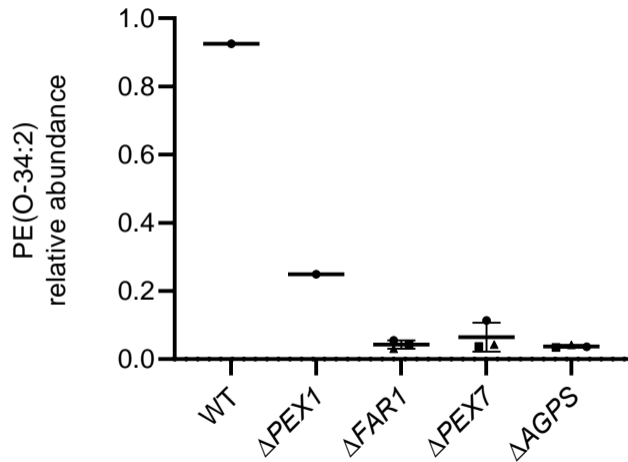

B

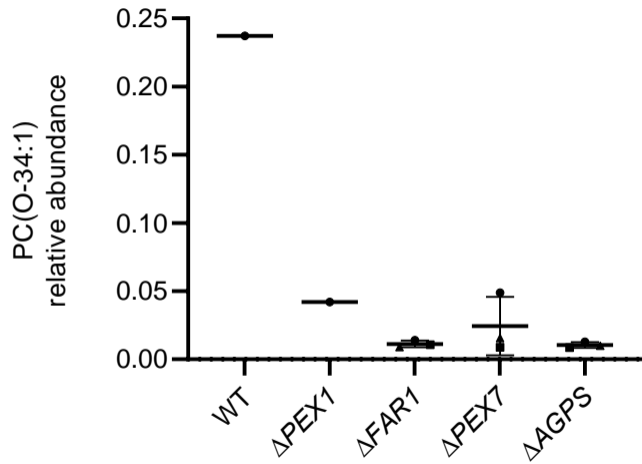

Supplement: Figure S2 [file mmc2.pdf]

A

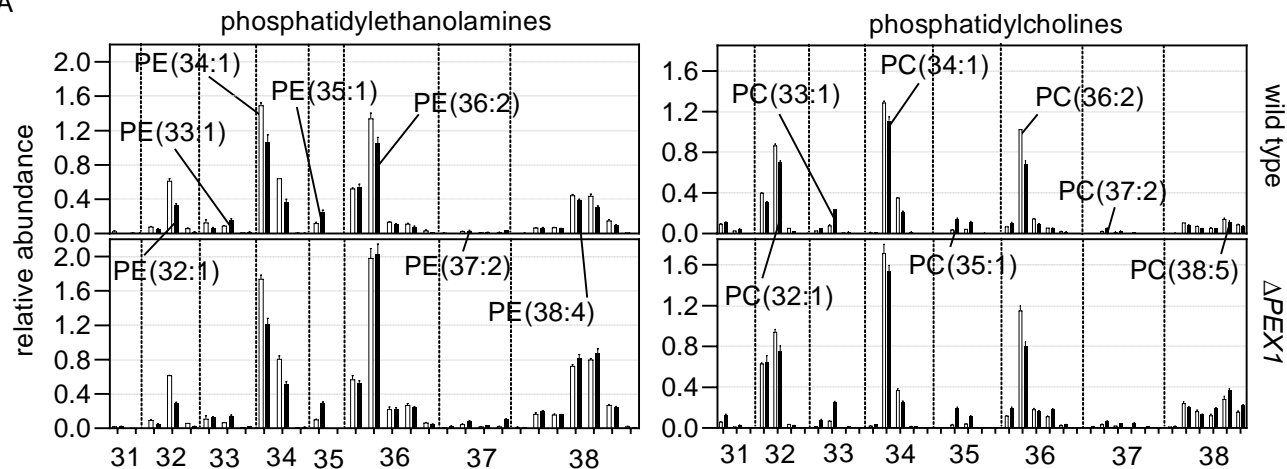

B

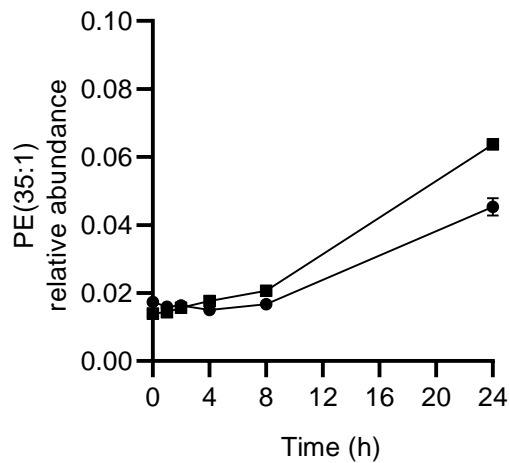

C

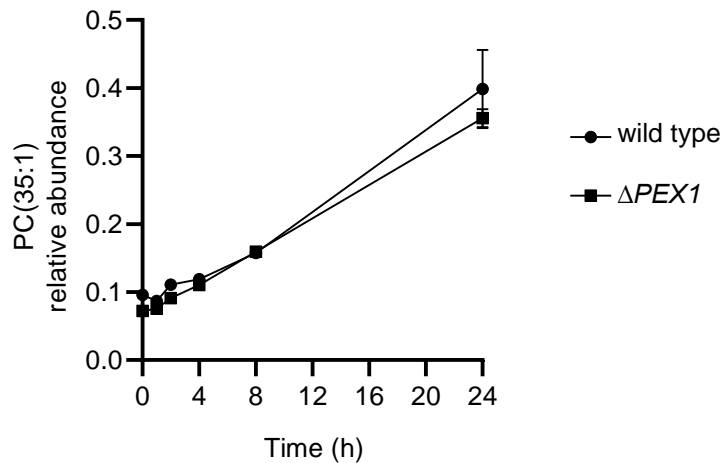

Supplement: Figure S3 [file mmc3.pdf]

A

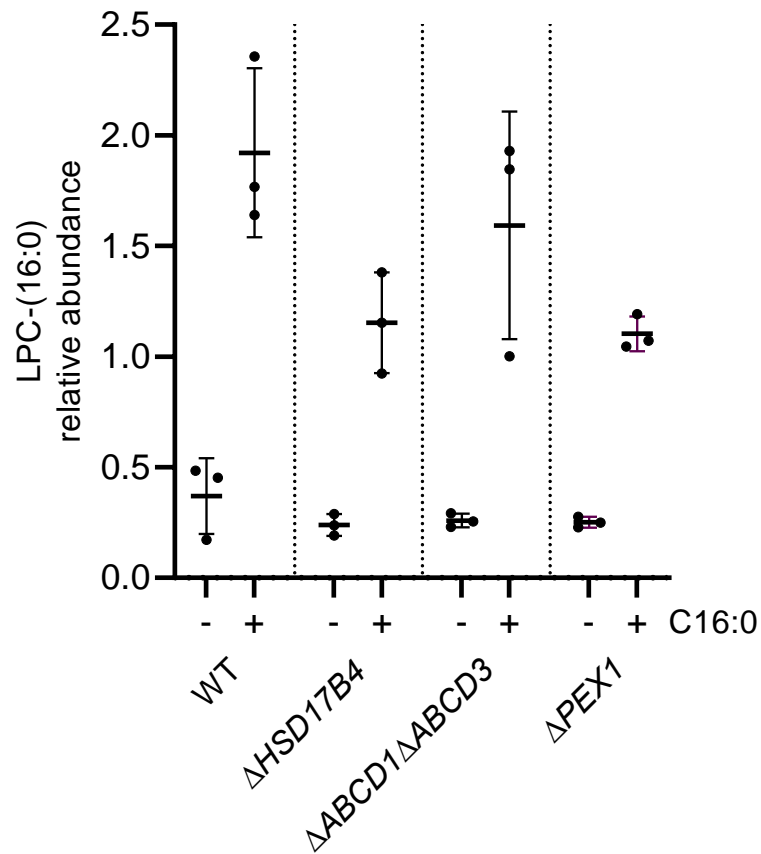

B

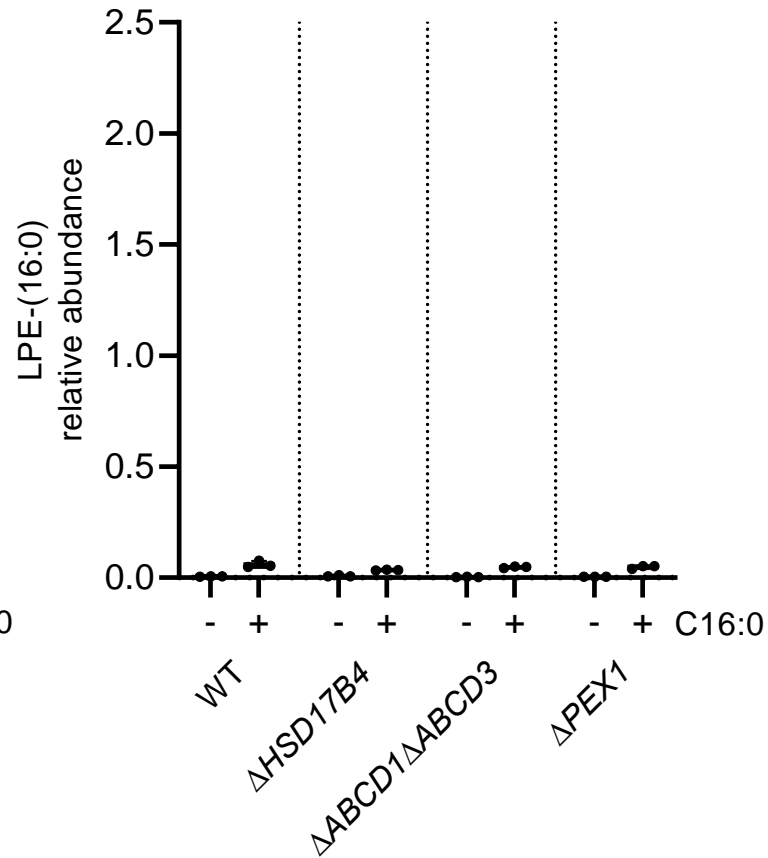

Supplement: Figure S4 [file mmc4.pdf]

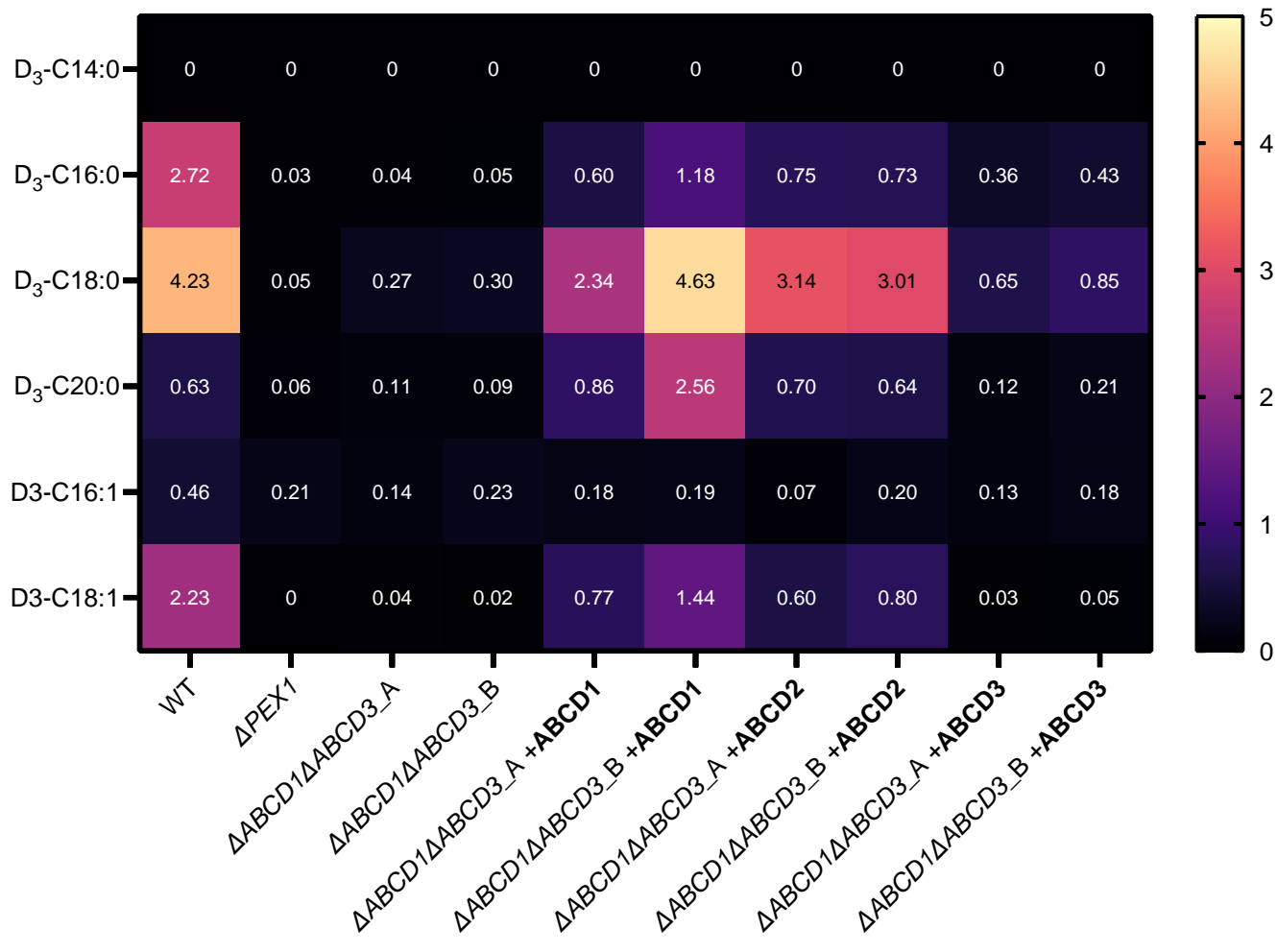

Supplement: Figure S5 [file mmc5.pdf]

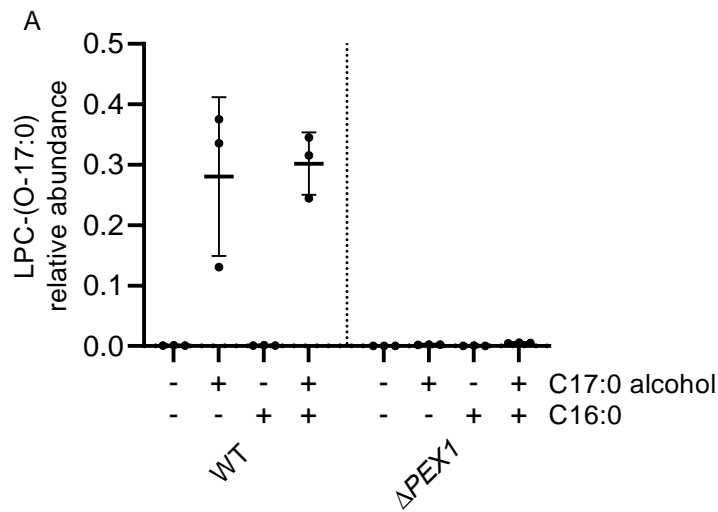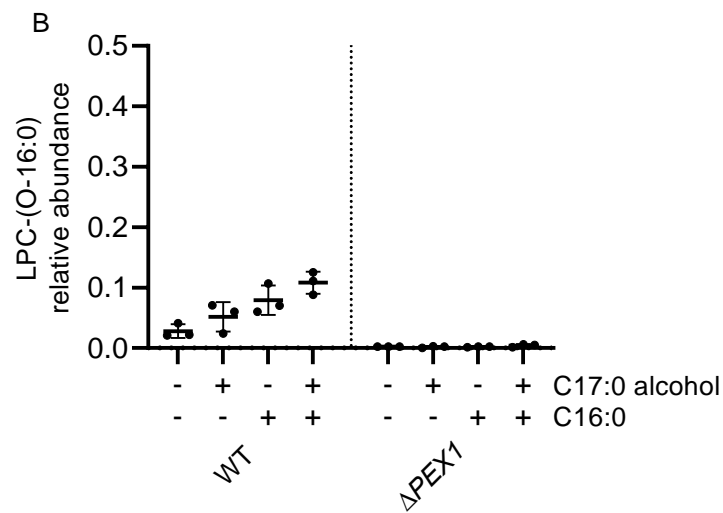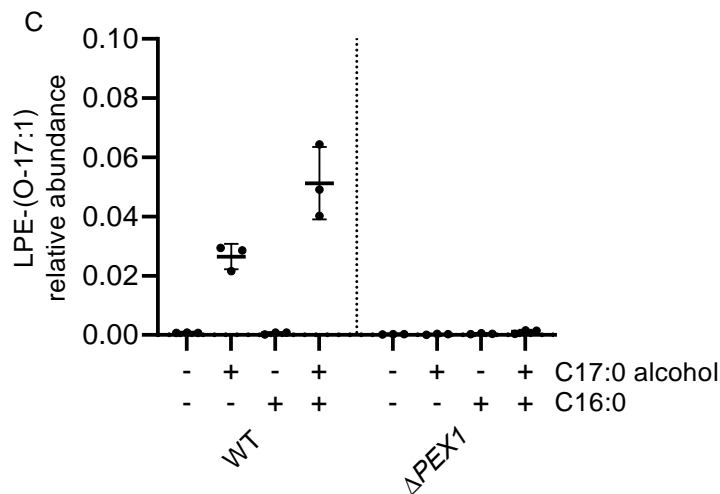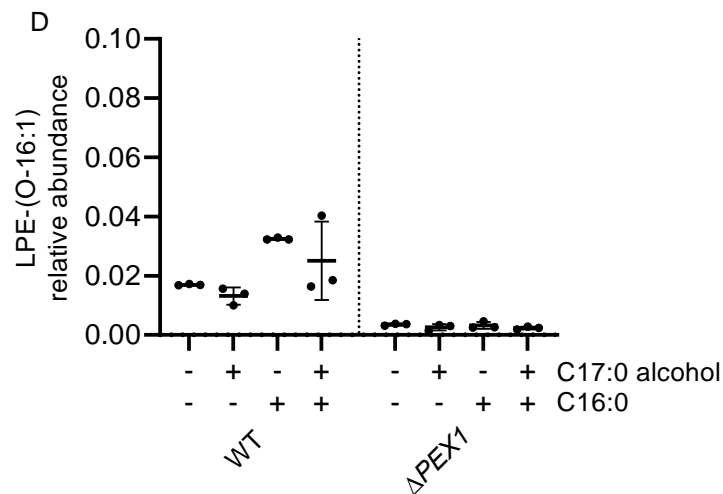

Supplement: Figure S6 [file mmc6.pdf]
