# Supplementary material for: The origin of long-chain fatty acids required for de novo ether lipid/plasmalogen synthesis
Source: J Lipid Res. 2023 Mar 28;64(5):100364. doi: 10.1016/j.jlr.2023.100364 (PMC10154978; doi:10.1016/j.jlr.2023.100364)
Supplement: supplemental figure captions [file mmc7.docx]

Supplemental Figure Legends for **The origin of long-chain fatty acids required for** ***de novo* ether lipid/plasmalogen synthesis**

Supplemental Figure 1. Extended results of Figure 1. Full profiles of phosphatidylethanolamine and phosphatidylcholine ether lipids in wild type and ∆*PEX1* HeLa cells. Cells were cultured under standard conditions with DMSO (white bars) or 40 µM of 1-heptadecanol (black bars) for 24 hours. Relative abundance of lipids is defined as relative to the corresponding internal standards added prior to lipid extraction (arbitrary units) (see Materials and Methods section). The results are shown as mean ± standard deviation (n = 2).

Supplemental Figure 2. Relative abundance of PE(O-34:2) (A) or PC(O-34:1) (B) ether phospholipids in wild type, ∆*PEX1*, ∆*FAR1*, ∆*PEX7*, or ∆*AGPS* HeLa cells. Relative abundance of lipids is defined as relative to the corresponding internal standards added prior to lipid extraction (arbitrary units) (see Materials and Methods section). The data show one experiment in three different single-cell clones (plotted with different symbols) and mean ± standard deviation.

Supplemental Figure 3. The relative abundance of non-ether phospholipids with an odd number of side chain carbons after incubation of cells with 1-heptadecanol. (A) Profiles of phosphatidylethanolamine (PE) and phosphatidylcholine (PC) non-ether lipids in wild type and ∆*PEX1* HeLa cells. Cells were cultured with (black bars) or without (white bars) 1-heptadecanol. Non-ether phospholipids with side chains comprising a total of 31 to 38 carbon atoms and a different number of double bonds (increases from left to right) are presented. The results are shown as mean ± standard deviation (n = 2). (B, C) Time-dependent formation of PE(35:1) and PC(35:1) in wild type or ∆*PEX1* HeLa cells after incubation with 1-heptadecanol. The results are shown as mean ± standard deviation (n = 3). Relative abundance of lipids is defined as relative to the corresponding internal standards added prior to lipid extraction (arbitrary units) (see Materials and Methods section).

Supplemental Figure 4. Synthesis of non-ether hexadecanoyl chain-containing (lyso)phosphatidylcholine (A) and (lyso)phosphatidylethanolamine (B) after incubation with hexadecanoic acid. Wild type, ∆*HSD17B4*, ∆*ABCD1*∆*ABCD3*, or ∆*PEX1* cells were cultured with 1-heptadecanol (40µM) and in addition with or without hexadecanoic acid (100 µM), as indicated. The results are shown as individual values and mean ± standard deviation (n = 3). Relative abundance of lipids is defined as relative to the corresponding internal standards added prior to lipid extraction (arbitrary units) (see Materials and Methods section).

Supplemental Figure 5. Extended results of Figure 7. Wild type, ∆*PEX1*, ∆*ABCD1*∆*ABCD3*, or ∆*ABCD1*∆*ABCD3* HeLa cells after expression of one of the ABCD transporters, were incubated with 30 µM of D_3_-docosanoic acid for 24 hours. The abundance of deuterium-labeled acids (µmol/g of protein) was measured, the results are shown as mean values and false-colored accordingly to the color scale bar shown on the right side (n = 3).

Supplemental Figure 6. Synthesis of 1-heptadecanol-containing (A, C) or 1-hexadecanol-containing (B, D) ether (lyso)phosphatidylcholines (A, B) and ether (lyso)phosphatidylethanolamines (C, D). Wild type or ∆*PEX1* cells were cultured with 1-heptadecanol (40µM) and in addition with or without hexadecanoic acid (100 µM), as indicated. The results are shown as individual values and mean ± standard deviation (n = 3). Relative abundance of lipids is defined as relative to the corresponding internal standards added prior to lipid extraction (arbitrary units) (see Materials and Methods section).
